# Hospital physician awareness about management of antibiotic-resistant gram-negative (ARGN) infections

**DOI:** 10.1186/2197-425X-3-S1-A128

**Published:** 2015-10-01

**Authors:** P Irani, R Epstein, RE Aubert, M Khalid, E Epstein, JR Teagarden, T Salimi

**Affiliations:** AstraZeneca, Global Medicines Department, London, United Kingdom; Epstein Health, LLC, Woodcliff Lake, United States; Research and Evaluation Analytics, LLC, Ringwood, United States; AstraZeneca, Global Medical Affairs, Gaithersburg, United States

## Introduction

New antibiotics have been introduced over the years to overcome drug resistance, but even these antibiotics have lost their efficacy against important Gram-negative bacteria with resistance to these drugs increasing rapidly across Europe and adjacent regions. We surveyed 800 hospital-based physicians in 4 European countries and Russia to assess their level of ARGN awareness and perceived ability to manage this growing and serious problem.

## Objectives

· Assess hospital-based physician awareness about management of ARGN infections

· Determine factors influencing physician awareness of ARGN infections

· Identify opportunities to improve the diagnosis and management of ARGN infections

## Methods

We selected 800 hospital-based physicians from an online panel of 7,700 physicians who reside in Italy, Spain, France, Germany, or Russia, and who all have recent experience in the treatment of patients with Gram-negative bacterial infections. They answered questions through a secured, online web-based survey system. We used a multivariate analysis to identify factors independently affecting physician awareness of how best to manage ARGN infections.

## Results

Of participating physicians, 55% are in academic/university hospitals while 38% are in community hospitals. About 45% have practiced >15 years, and the same proportion reported treating >5 patients with ARGN infections in the prior month. Less than a third of respondents felt either very or extremely aware of how best to treat patients with ARGN infections, yet 64% believe their colleagues are aware of best practices and 75% believe their hospitals are prepared to treat these infections. Only half of the respondents believe guidelines exist in their hospitals for treating ARGNs and about half believe rapid molecular diagnostic tests are available to them. While 75% report using clinical decision support tools generally, most do not have access to a specific tool to predict those at high risk for ARGN infections; 86% said they consider such a tool necessary. More years in practice, recent experience treating a high volume of ARGN infections, availability of local treatment guidelines, specialization in infectious diseases, and practicing in academic and university treatment centers were independently associated with greater physician awareness.

## Conclusions

A high proportion of hospital-based physicians in the 5 countries covered by the survey consider themselves unaware of the best ways to manage ARGN infections. Various factors affect the degree of their awareness, but in general, they indicate a need for more support specifically through enhanced availability of guidelines, rapid molecular tests, prediction tools, and effective antibiotics.

## Grant Acknowledgment

AstraZeneca funded the survey research.

